# RACK1 promotes lung cancer cell growth via an MCM7/RACK1/Akt signaling complex

**DOI:** 10.18632/oncotarget.17120

**Published:** 2017-04-15

**Authors:** Liangru Fei, Yinan Ma, Meiyu Zhang, Xiaofang Liu, Yuan Luo, Congcong Wang, Haiyan Zhang, Wenzhu Zhang, Yuchen Han

**Affiliations:** ^1^ Department of Pathology, School of Basic Medical Sciences, China Medical University, Shenyang 110000, China; ^2^ Department of Pathology, The First Affiliated Hospital of China Medical University, Shenyang 110000, China; ^3^ Department of Pathology, The First People's Hospital of Jining, Shandong 272000, China

**Keywords:** RACK1, MCM7, proliferation, NSCLC, Akt

## Abstract

MCM7, a member of the miniature chromosome maintenance (MCM) protein family, is crucial for the initiation of DNA replication and proliferation in eukaryotic cells. In this report, we demonstrate that RACK1 regulates cell growth and cell cycle progression in human non-small-cell lung cancer by mediating MCM7 phosphorylation through an MCM7/RACK1/Akt signaling complex. RACK1 functions as a central scaffold that brings Akt into physical proximity with MCM7. Overexpression of RACK1 increases interactions between Akt and MCM7 and promotes Akt-dependent MCM7 phosphorylation, which in turn increases MCM7 binding to chromatin and MCM complex formation. Together, these changes promote DNA replication and cell proliferation. Our findings reveal a novel signaling pathway that regulates growth in non-small cell lung cancer.

## INTRODUCTION

A heterohexameric complex composed of MCM2-7, which acts as a DNA replication ligase with helicase activity at replication forks, initiates chromosomal duplication, a highly precise event that maintains genome stability [1–6]. MCM proteins 2–7, which are highly conserved between yeast and humans, are recruited to chromatin and bind with origin recognition complex (ORC), Cdt1, and cdc6 to form the pre-replication complex (Pre-RC) early during the G1 phase, ultimately initiating DNA replication [7, 8]. Moreover, numerous studies have demonstrated that MCM7 is a pivotal component of the DNA replication initiation complex in *Xenopus* and yeast [9–12]; it also serves as a proliferation marker and is correlated with tumorigenesis in several human malignancies, including prostate cancer [13], ovarian cancer [14], endometrial carcinoma [15], oral squamous cell carcinoma [16], esophageal adenocarcinoma [17], colorectal adenocarcinoma [18], and glioblastoma [19]. In addition, MCM7 is associated with mRNA transcription and DNA damage [20–22]. Recent studies have demonstrated that MCM7 is a potential therapeutic target in several cancers [13, 23–25].

Receptor for activated C kinase 1 (RACK1) is a highly-conserved WD40 repeat scaffold protein that belongs to the Trp-Asp (WD) repeat protein family. Individual WD40 repeats can simultaneously interact with multiple signaling molecules, including PKC [26], Src [27–29], integrin [30], EphB3 [31], and c-Abl [32], which allows RACK1 to integrate inputs from various signaling pathways [33]. RACK1 therefore plays a pivotal role in many critical cellular processes. Activation of Akt, a Ser/Thr kinase that participates in many cellular processes by facilitating growth factor-mediated cell survival and blocking apoptosis [34], is associated with tumorigenesis in various human cancers. In addition, a recent study in NSCLC revealed that P-Thr308, but not P-Ser473, which is widely used as a marker of Akt activity, is the major regulator of Akt protein kinase activity [35].

Here, we found that RACK1 was up-regulated in NSCLC, and knockdown of RACK1 inhibited cellular growth and blocked S phase entry. Furthermore, we demonstrated that the oncogenic potential of RACK1 was correlated with MCM7 function. RACK1 regulated the recruitment of MCM7 to chromatin and its interaction with other MCM proteins by regulating its phosphorylation via an MCM7/RACK1/Akt signaling complex. These results suggest that RACK1 promotes growth in NSCLC by facilitating interactions between MCM7 and Akt.

## RESULTS

### RACK1 promotes cellular proliferation by regulating G1/S progression in NSCLC cells

**Figure 1:**
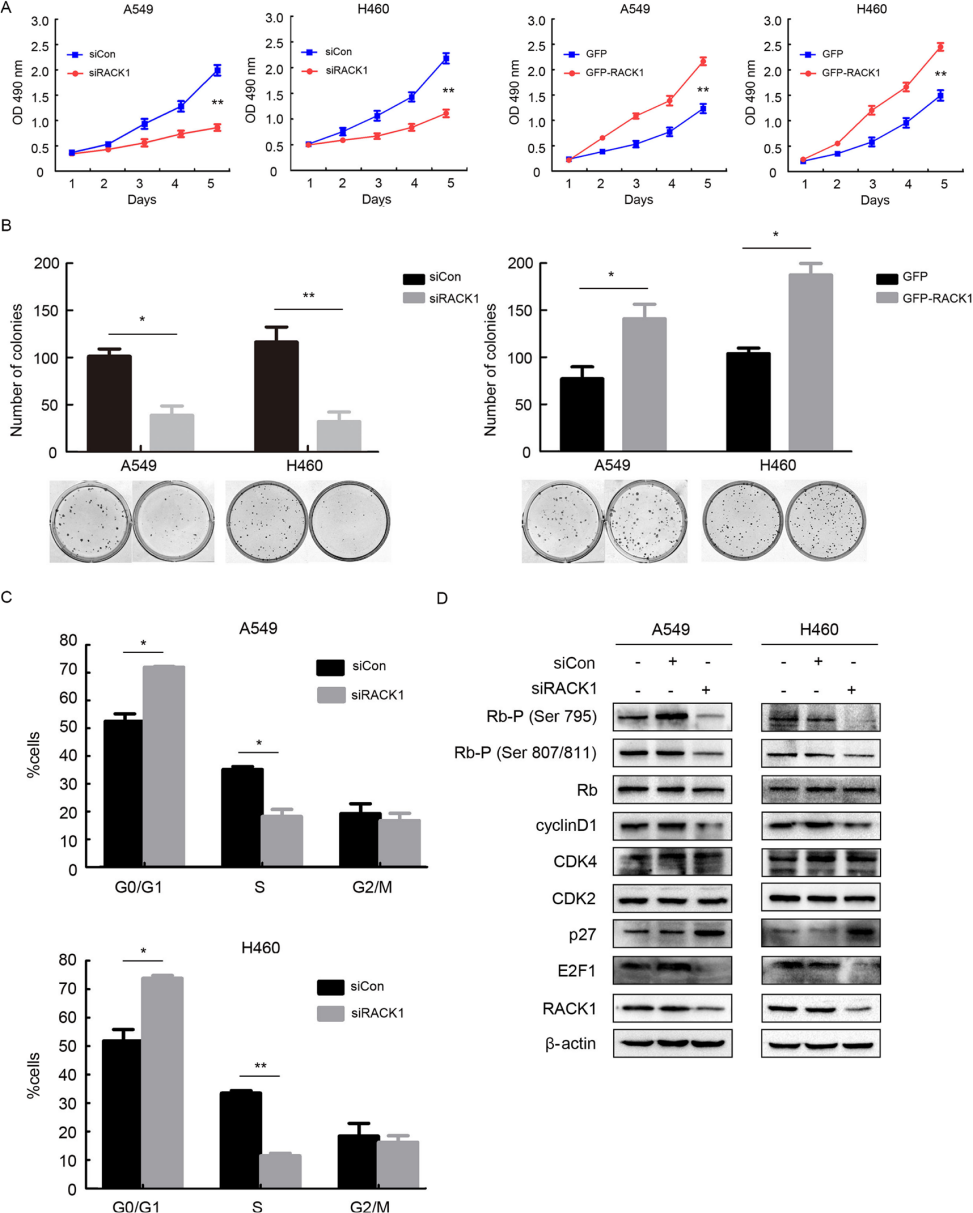
RACK1 promotes cellular proliferation by regulating G1/S progression in NSCLC cells A549 and H460 cell lines were transfected with siRNA RACK1 (siRACK1), siRNA control (siCon), pEGFP-N1-RACK1 (GFP-RACK1), or pEGFP-N1 (GFP) as indicated. (**A**) MTT assays for A549 and H460 siRACK1, siCon, GFP-RACK1, and GFP cells. (**B**) A549 and H460 cells were plated in 40-mm dishes 24 h after transfection and cultured in media supplemented with 10% FBS for 12 days, after which the number of colonies with more than 50 cells was counted. (**C** and **D**) A549 and H460 cells were synchronized at the G0/G1 phase by serum starvation, cell cycle progression was then triggered by the addition of 10% FBS for 4h, and flow cytometry (C) and the activity of G1 cell cycle regulators (D) were analyzed to evaluate cell cycle progression.

To understand the function of RACK1 in NSCLC cells, we used siRACK1 to knock down its expression in the A549 and H460 NSCLC cell lines. RACK1 knockdown inhibited, while RACK1 overexpression promoted, cell growth and colony formation (Figure 1A and 1B). Furthermore, flow cytometry revealed that RACK1 knockdown effectively blocked entry into S-phase and reduced the percentage of cells in S-phase, suggesting that RACK1 might regulate the G1 checkpoint (Figure 1C). To confirm this, we examined the effects of RACK1 on regulators of cell cycle progression at the G1/S boundary. Downregulation of RACK1 decreased cyclinD1 levels, induction of the CDK inhibitor p27, dephosphorylation of Rb, and sequestration of the transcription factor E2F1, but did not alter CDK2, CDK4, or Rb expression, in G1 cells compared to negative controls (Figure 1D).

### RACK1 interacts with MCM7

**Figure 2:**
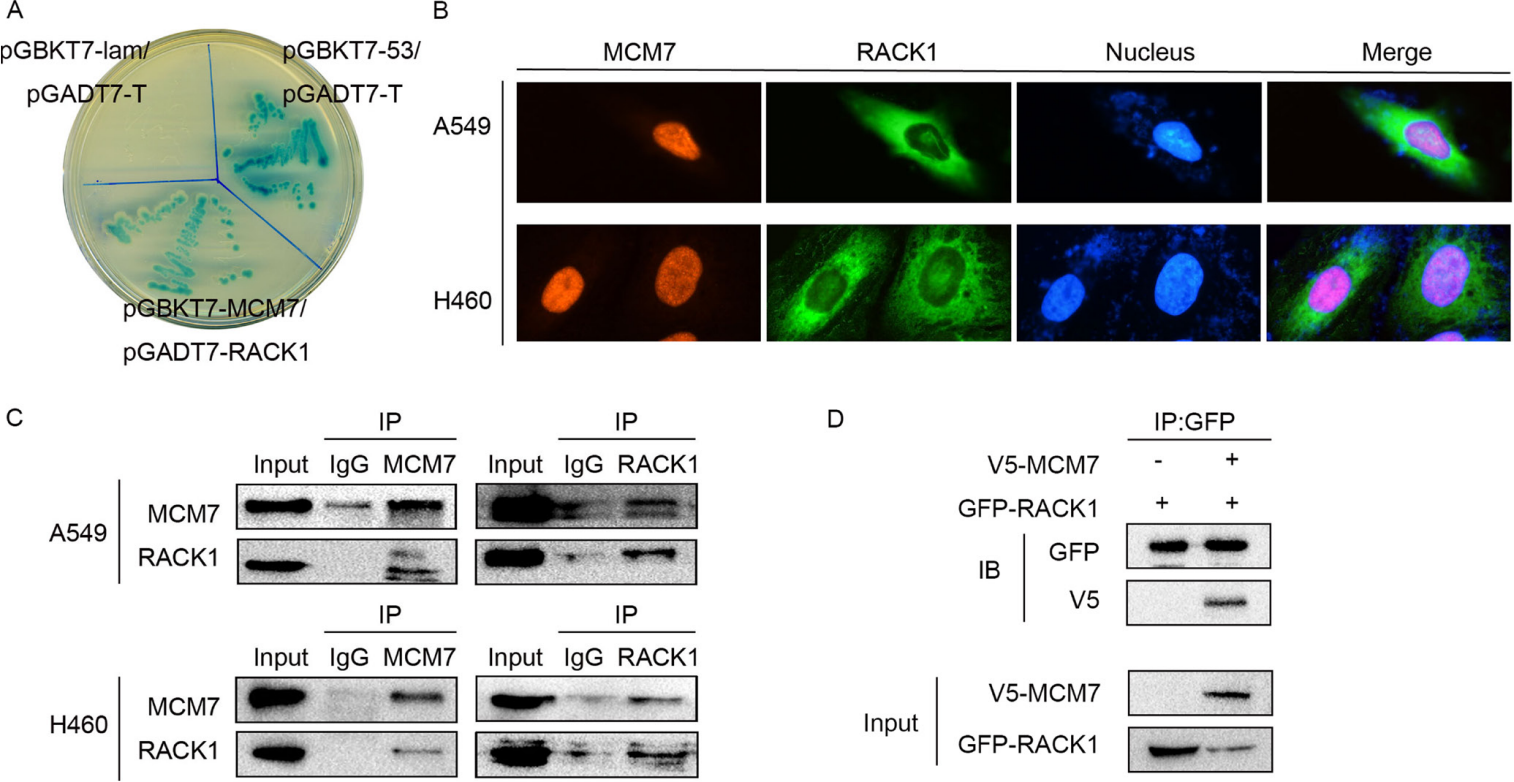
RACK1 interacts with MCM7 (**A**) pGBKT7-MCM7 and pGADT7-RACK1 co-transformants were grown on SD agar plates with highly stringent nutrient selection (SD-Leu-Trp-His-Ade). pGBKT7-p53 and pGADT7-T-antigen co-transformants were the positive control and pGBKT7-lam and pGADT7-T-antigen co-transformants were the negative control. (**B**) Immunofluorescence staining of A549 and H460 cells with anti-RACK1 primary and anti-mouse FITC-conjugated secondary antibodies and with anti-MCM7 primary and anti-rabbit TRITC-conjugated secondary antibodies. (**C**) Co-immunoprecipitation (IP) of RACK1 (left) or MCM7 (right) in A549 and H460 cells. The immunoprecipitates were immunoblotted (IB) with the indicated antibodies. (**D**) A549 cells were transfected with GFP-RACK1 and V5-MCM7, cell lysates were immunoprecipitated with GFP antibody, and immunoblotting was performed with GFP or V5 antibody.

RACK1 is a scaffold protein that is able to interact with several signaling molecules simultaneously [36]. A two-hybrid yeast assay revealed that RACK1 bound with MCM7, which was a potential downstream regulator of G1/S transition in NSCLC (Figure 2A). Double immunofluorescence staining in A549 and H460 cells indicated that RACK1 was mainly localized in the cytoplasm but was also expressed to a lesser degree in the nucleus together with MCM7 (Figure 2B). Both endogenous (Figure 2C) and exogenous (Figure 2D) co-immunoprecipitation of RACK1 and MCM7 confirmed their interaction.

### RACK1 and MCM7 expression are elevated in clinical NSCLC samples

**Figure 3:**
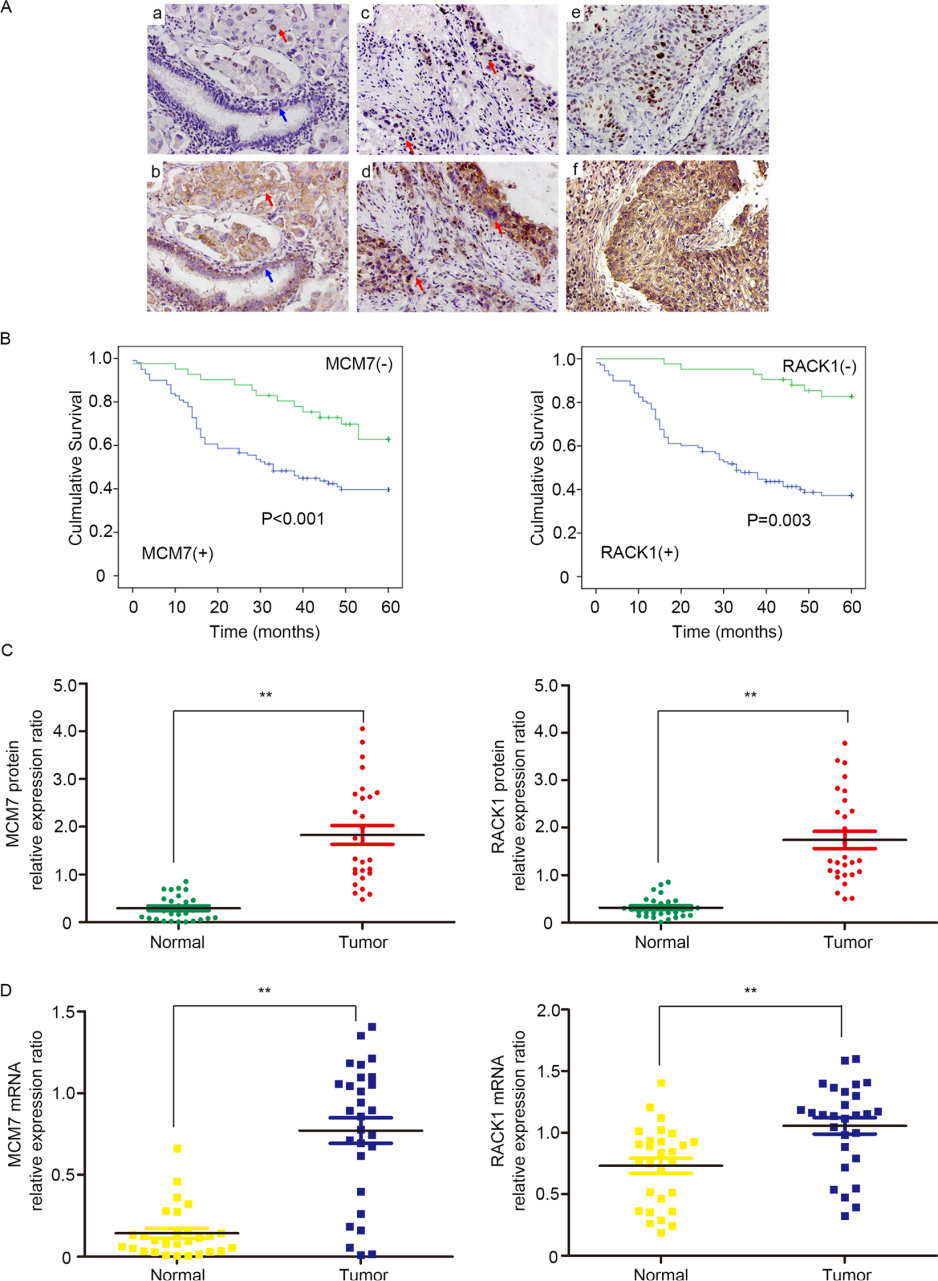
RACK1 and MCM7 expression are elevated in clinical NSCLC samples (**A**) Immunohistochemistry was used to examine RACK1 and MCM7 expression in NSCLC. a, Nuclear MCM7 expression in poorly-differentiated adenocarcinoma of the lung (red arrow) and negative expression in normal bronchial epithelium (blue arrow). b, Cytoplasmic RACK1 expression in poorly-differentiated adenocarcinoma of the lung (red arrow) and in normal bronchial epithelium (blue arrow). c, MCM7 expression in *in situ* carcinoma ( upper red arrow) and in poorly-differentiated squamous cell carcinoma of the lung (lower red arrow). d, RACK1 expression in *in situ* carcinoma (upper red arrow) and in poorly-differentiated squamous cell carcinoma of the lung (lower red arrow). e, MCM7 expression in poorly-differentiated squamous cell carcinoma of the lung. f, RACK1 expression in poorly-differentiated squamous cell carcinoma of the lung. (**B**) Overall survival rates in 150 NSCLC patients were compared between low and high MCM7 (left) and RACK1 (right) level groups using the Kaplan-Meier method. (**C**) Western blot analysis of MCM7 (left) and RACK1 (right) expression in NSCLC and paired noncancerous tissues. β-actin protein level was quantified as an internal control. (**D**) RT-PCR results showing relative expression of MCM7 (left) and RACK1 (right) in NSCLC and paired noncancerous tissues. β-actin mRNA expression was quantified as an internal control.

Next, we performed immunohistochemical staining for the RACK1/MCM7 complex in NSCLC specimens. RACK1 and MCM7 expression were higher in *in situ* carcinoma and cancer cells than in normal bronchial epithelium cells (Figure 3A). We then performed immunohistochemical analysis of 150 NSCLC samples using tissue chips and found that RACK1 levels were positively correlated with MCM7 levels (Table 1). Moreover, both RACK1 and MCM7 levels were positively correlated with histological grade, lymphatic metastasis, and tumor TNM stage (Table 2). A log-rank test showed that NSCLC patients with high RACK1 and MCM7 levels had shorter overall survival (Figure 3B). To further confirm these findings, we assessed RACK1 and MCM7 mRNA and protein levels in an additional 28 pairs of matched NSCLC and normal lung tissue samples. mRNA and protein levels of both RACK1 and MCM7 were higher in tumor tissue than in the normal lung counterparts (Figure 3C and 3D). Taken together, these findings indicate that RACK1 and MCM7 are important promoters of NSCLC development and progression.

**Table 1:** Correlation between RACK1 and MCM7 expression in NSCLC

MCM7 expression						
		Positive		Negative		
			*N*		R	*P* value
RACK1 expression	Positive	89		19	0.555	< 0.001
	Negative	10		32		

**Table 2:** Relationship between RACK1 and MCM7 expression and clinical and pathological features in NSCLC patients

Clinical characteristics	No. patients	MCM7-positive	*P* value	RACK1-positive	*P* value
Age					
< 55	22	14	0.811	15	0.797
≥ 55	128	85		93	
Gender					
Male	118	81	0.130	84	0.664
Female	32	14		21	
Histological grade					
I–II	89	39	0.012	44	0.020
III	61	40		46	
Lymphatic metastasis					
Negative	98	43	0.026	48	0.024
Positive	52	33		36	
TNM classification					
I–II	102	58	0.019	55	0.032
III–IV	48	37		35	

### RACK1 promotes phosphorylation of MCM7

**Figure 4:**
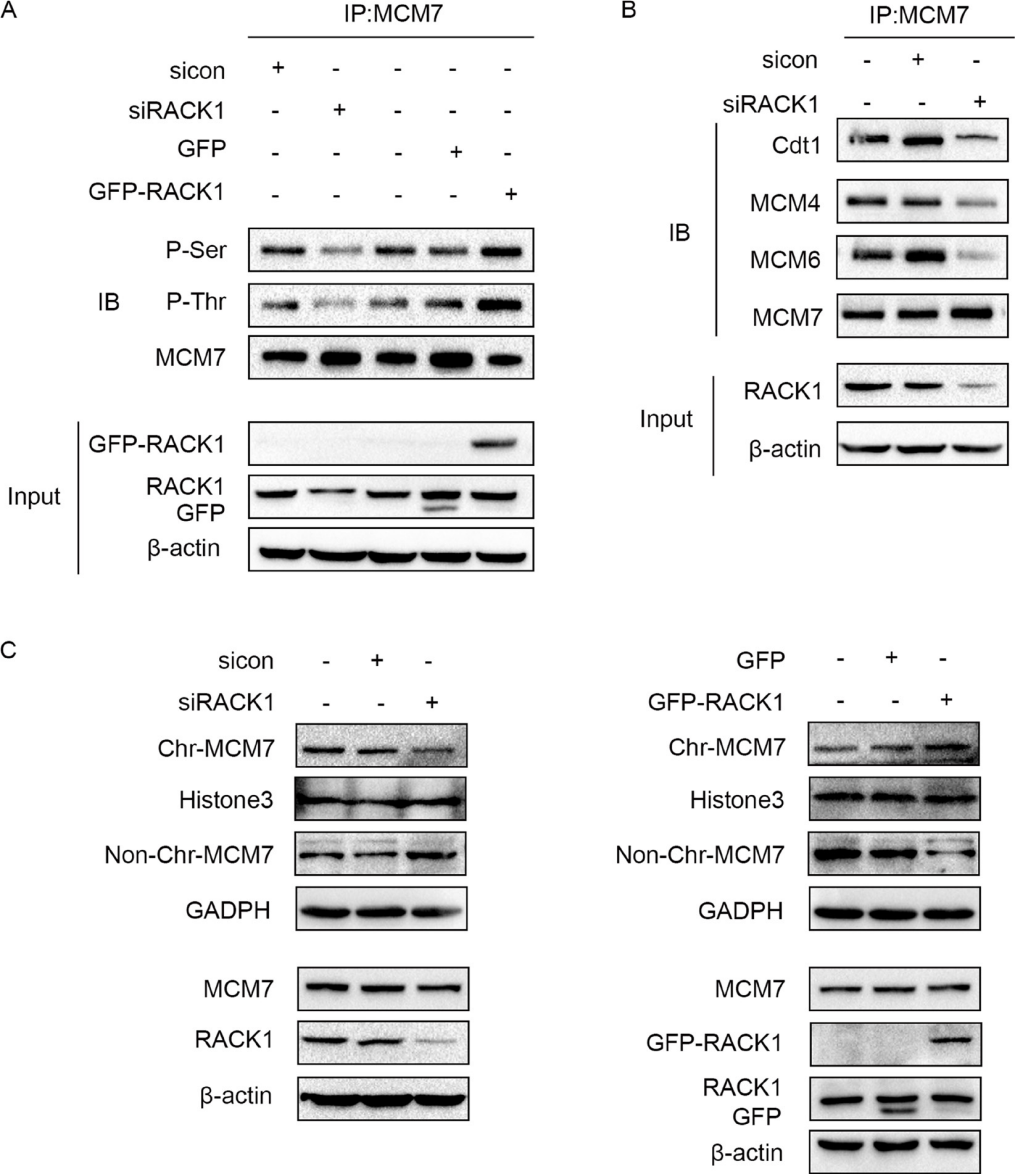
RACK1 promotes phosphorylation of MCM7 (**A**) A549 cells were transfected with siRACK1, siCon, GFP-RACK1, or GFP. MCM7 immunoprecipitates were probed for P-Ser, P-Thr, and MCM7 as indicated. (**B**) A549 cells were transfected with siRACK1 or siCon. MCM7 immunoprecipitates were probed for Cdt1, MCM4, MCM6, and MCM7. (**C**) A549 cells were treated with siRACK1, siCon, GFP-RACK1, or GFP as indicated. The chromatin (Chr) and non-chromatin (Non-Chr) fractions of these cells were purified and immunoblotted with anti-MCM7 antibodies. Antibodies against Histone 3 and GADPH were used as internal controls.

We then examined the mechanism by which RACK1 interacts with MCM7. Interestingly, RACK1 promoted the phosphorylation of MCM7 (Figure 4A), which increases its activity [37–39]. As expected, phosphorylation of MCM7 increased its recruitment to chromatin and MCM complex formation (Figure 4B and 4C), which are both critical for DNA replication and cellular proliferation [40, 41]. These results suggest that RACK1 promotes cellular growth and cell cycle progression via phosphorylation of MCM7.

### RACK1 promotes MCM7 phosphorylation indirectly via the Akt signaling pathway

**Figure 5:**
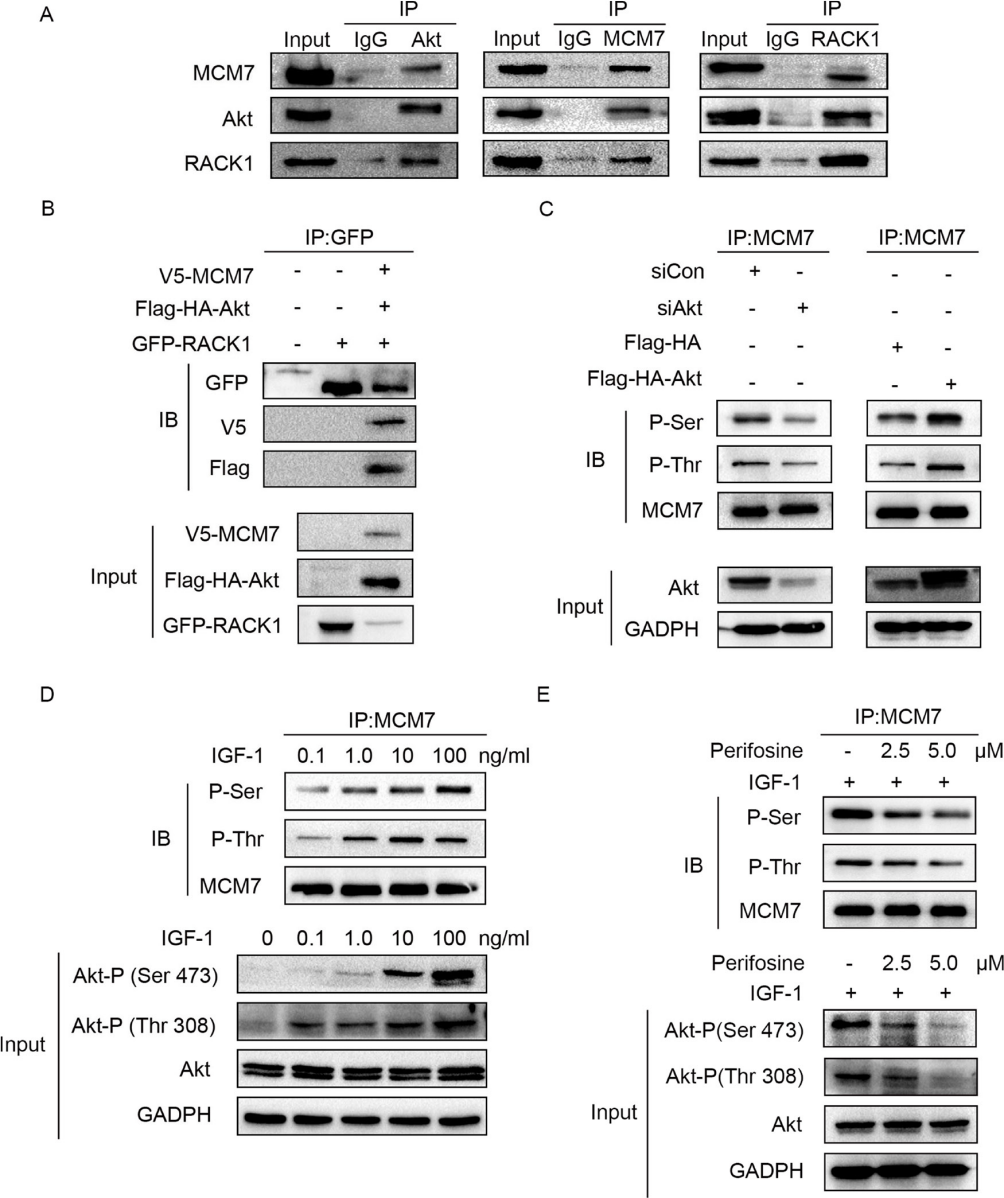
Akt regulates MCM7's phosphorylation (**A**) A549 cell lysates were immunoprecipitated with Akt, MCM7, or RACK1 antibody and then immunoblotted with the indicated antibodies. (**B**) A549 cells were transfected with GFP-RACK1 together with V5-MCM7 and Flag-HA-Akt. Cell lysates were immunoprecipitated with GFP antibody and then immunoblotted with GFP or V5 or Flag antibody. (**C**) A549 cells were transfected with siRNA Akt (siAkt), siRNA control (siCon), pcDNA-Flag-HA-Akt1 (Flag-HA-Akt), or pcDNA-Flag-HA-Akt1 (Flag-HA). MCM7 immunoprecipitates were probed for P-Ser, P-Thr, and MCM7. (**D**) A549 cells were serum-starved overnight and then treated with different doses of IGF-1 as indicated for 10 min. MCM7 immunoprecipitates were probed for P-Ser, P-Thr, and MCM7. (**E**) Serum-starved A549 cells were treated with 2.5 or 5.0 μM perifosine as indicated for 1 h prior to the addition of 100 ng/mL IGF-1 for 10 min. MCM7 immunoprecipitates were probed for P-Ser, P-Thr, and MCM7.

**Figure 6:**
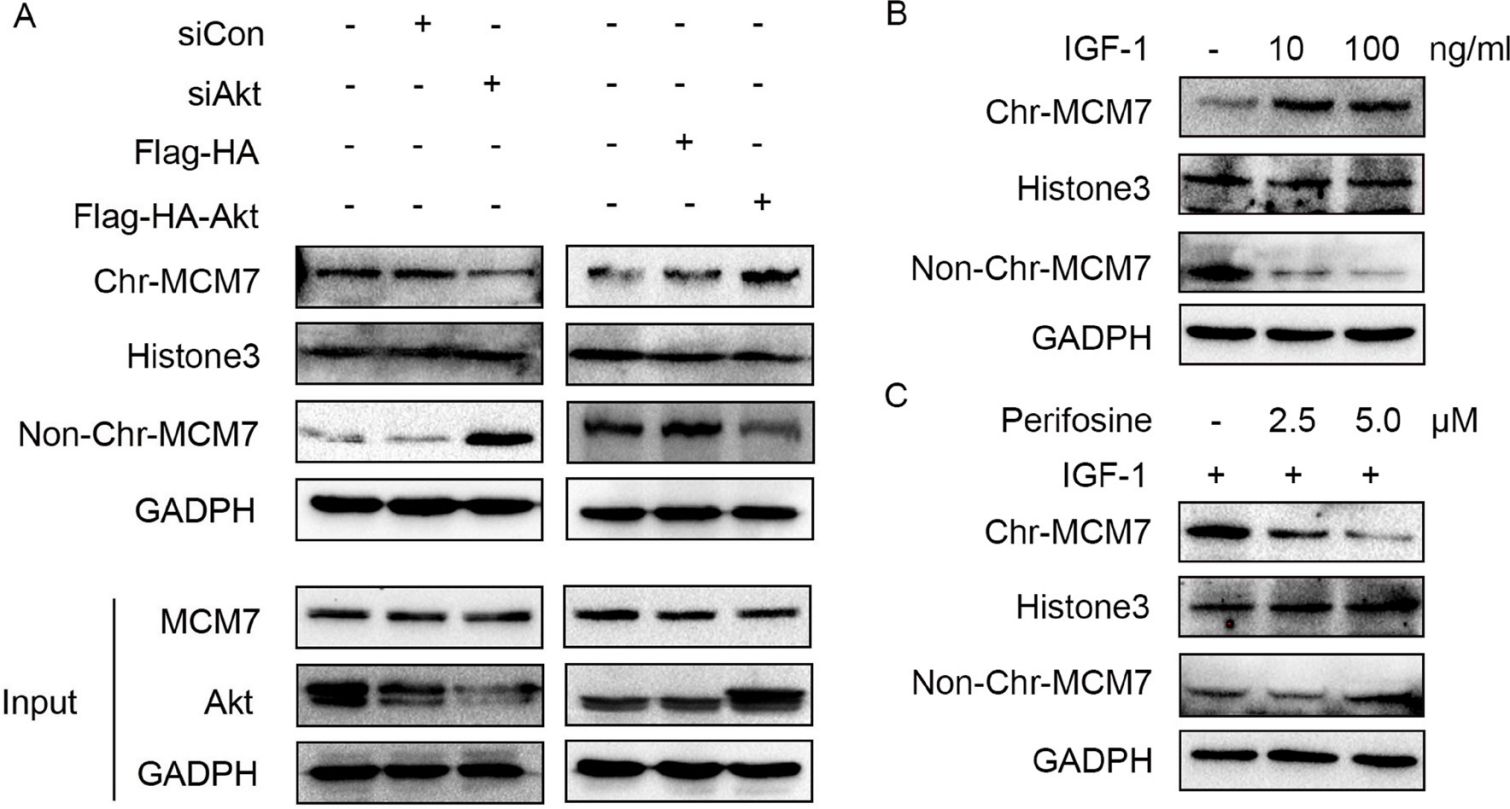
Akt regulates MCM7's loading on chromatin (**A**) A549 cells were transfected with siAkt, siCon, Flag-HA-Akt, or not treated. The chromatin (Chr) and non-chromatin (Non-Chr) fractions of these cells were purified and immunoblotted with anti-MCM7 antibodies. (**B**) A549 cells were serum-starved overnight and then treated with 10 or 100ng/mL IGF-1 for 10min as indicated. The chromatin (Chr) and non-chromatin (Non-Chr) fractions of these cells were purified and immunoblotted with anti-MCM7 antibodies. (**C**) Serum-starved A549 cells were treated with 2.5 or 5.0 μM perifosine as indicated for 1 h prior to the addition of 100 ng/mL IGF-1 for 10 min. The chromatin (Chr) and non-chromatin (Non-Chr) fractions of these cells were purified and immunoblotted with anti-MCM7 antibodies.

**Figure 7:**
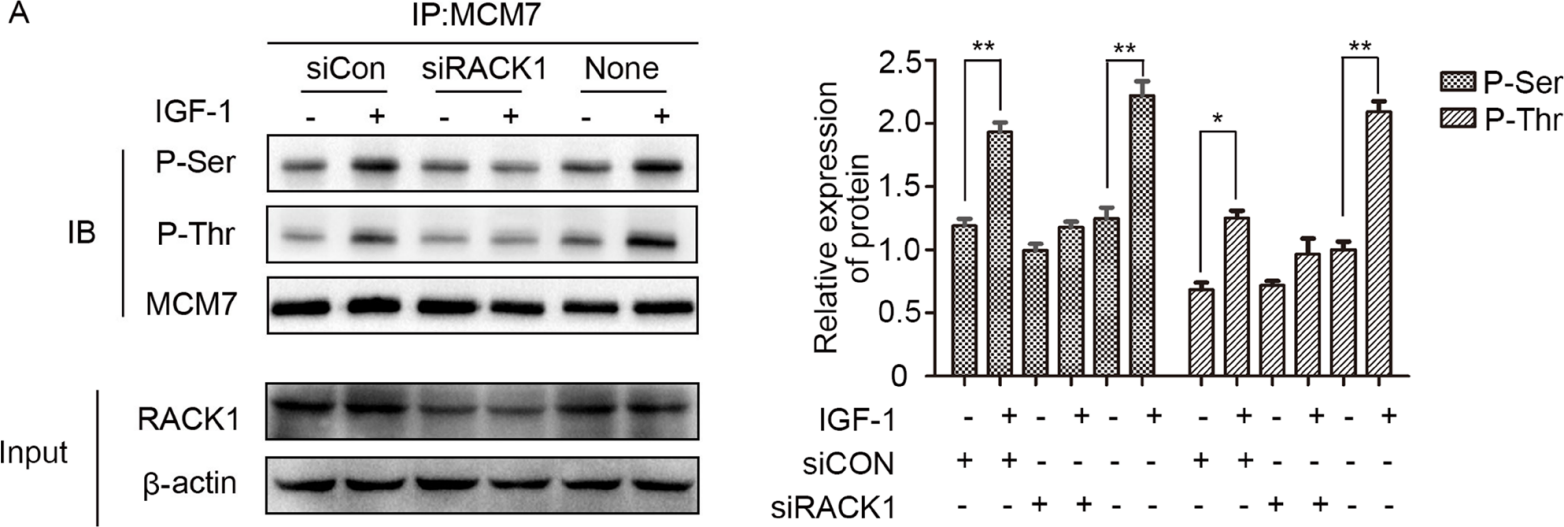
RACK1 is required for the interaction between Akt and MCM7 (**A)** A549 cells were transfected with siRACK1 or siCon, serum-starved, and then treated with 100 ng/mL IGF-1 for 10 min. MCM7 immunoprecipitates were probed for P-Ser, P-Thr, and MCM7.

Because RACK1 is a scaffold protein that does not contain catalytic domains, it likely promotes phosphorylation of MCM7 indirectly. Akt also interacted with RACK1 and MCM7; both endogenous (Figure 5A) and exogenous (Figure 5B) co-immunoprecipitation experiments confirmed the existence of a ternary MCM7/RACK1/Akt complex. Furthermore, Akt levels were positively correlated with MCM7 phosphorylation levels (Figure 5C–5E) and chromatin binding (Figure 6A–6C). These results suggest that RACK1 indirectly promotes MCM7 activation via the Akt signaling pathway. We therefore investigated whether Akt pathway-induced MCM7 serine/threonine phosphorylation was dependent on RACK1. RACK1 knockdown abolished Akt pathway-induced MCM7 phosphorylation (Figure 7A). RACK1 therefore plays an essential role by indirectly promoting MCM7 phosphorylation through the Akt signaling pathway.

## DISCUSSION

Here, we demonstrated that RACK1 regulates growth and cell cycle progression in human NSCLC by promoting MCM7 phosphorylation as a component of an MCM7/RACK1/Akt signaling complex. These findings describe a novel mechanism by which promotes proliferation in NSCLC.

Due to its ability to regulate reversible phosphorylation via interactions with protein kinases, phosphatases, and their substrates, RACK1 has been recognized as an important component of signal transduction in several pathways [42]. Overexpression of RACK1 served as a biomarker associated with increased pathological stage, tumor size, and lymph node involvement in pulmonary adenocarcinoma in a recent study [43]. In addition, RACK1 promotes NSCLC by interacting with and activating Smoothened, which in turn mediates Gli1-dependent transcription in NSCLC cells [44]. In this study, we identified a novel signaling pathway by which RACK1 together with MCM7 regulates cell growth and cell cycle progression in NSCLC. MCM7 has long been considered a vital component in the initiation of DNA replication. Numerous studies suggest that phosphorylation of MCM7 is directly correlated with its binding to chromatin and other MCM family members, and decreases in this binding inhibit the helicase activity of MCM7, DNA replication, S-phase entry, and cancer cell growth [38–40]. In addition, binding of TPA to PKC activates PKC and its binding to RACK1, which in turn triggers translocation of RACK1 from the nucleus to the cytoplasm, inactivation of MCM7, and disintegration of the DNA replication initiation complex in prostate cancer [41]. However, the specific mechanism by which RACK1 regulates MCM7 activity remains unclear. Here, we found that RACK1 functioned as a central scaffold that brought Akt and MCM7 into close physical proximity in NSCLC cells. Knockdown of RACK1 inhibited the interaction between Akt and MCM7 and decreased Akt-induced phosphorylation of MCM7, resulting in dissociation of MCM7 from chromatin and destruction of the MCM complex. Moreover, RACK1 knockdown inhibited DNA replication and S-phase entry by increasing cell cycle arrest at the G1/S transition checkpoint. In contrast, RACK1 overexpression had the opposite effects. It has also been reported that RACK1 promotes growth in other cancers by affecting key cell cycle regulators. Li *et al*. reported that downregulation of RACK1 decreased Cyclin D1 expression and induced G1/S cell cycle arrest in pancreatic ductal adenocarcinoma [45]. Lv *et al*. found that knockdown of RACK1 suppressed Cyclin D1 and CDK6 expression, thereby dramatically increasing G0/G1 phase and reducing S phase cell populations, in glioma [46]. Zhang *et al*. reported that stable RACK1 knockdown downregulated Cyclin B1 and Cyclin D1 and promoted G1 and G2 phase arrest in oral squamous cell carcinoma [47]. Interestingly, RACK1 has the opposite effect in colon cancer cells; RACK1 overexpression delays passage of HT-29 cells through G1 and mitotic checkpoints by suppressing Src-mediated Sam68 phosphorylation and maintaining the active state of CDK1-cyclinB [48, 49].

In contrast to previous studies, we found here that, although RACK1 was mainly localized in the cytoplasm, it was also expressed to a lesser degree in the nucleus. It has been reported that cAMP/PKA pathway activation may promote the translocation of RACK1 from the cytoplasm to the nucleus [50–53]. In addition, previous studies have obtained contradictory results regarding the role of RACK1 in regulating Akt activity in different cell types [31, 42, 54–56]. Our results indicate that RACK1 functions as a protein scaffold that forms a complex by dynamically recruiting MCM7 and Akt. However, there may be other mechanisms by which RACK1 regulates the Akt signaling pathway. In addition, we found that MCM7 phosphorylation was positively correlated with Akt signaling pathway activity in the presence of RACK1, as the MCM7 phosphorylation level was consistent with Akt activity resulting from enhanced IGF-1 stimulation. However, because MCM7 was not confirmed as a direct substrate of Akt, the specific phosphorylation sites and mechanism remain unclear. It is possible that MCM7 phosphorylation is regulated by another protein that also interacts with the Akt signaling pathway. Regardless, immunohistochemical analysis demonstrated that RACK1 expression was positively correlated with MCM7 expression and promoted tumorigenesis in NSCLC.

In conclusion, our results suggest that the scaffold protein RACK1 promotes progression in NSCLC progression by indirectly activating MCM7.

## MATERIALS AND METHODS

### Cell culture and synchronization

Human A549 lung adenocarcinoma cells and H460 large cell lung cancer cells (cell resource center of Shanghai Institutes for Biological Sciences, Chinese Academy of Sciences) were cultured in RPMI 1640 medium supplemented with 10% fetal bovine serum (FBS). For synchronization, cells were starved in medium without serum for 48 h.

### Antibodies and reagents

Antibodies for Akt, Akt-Ser(P)-473, Akt-Thr(P)-308, Rb, Rb-Ser(P)-807/811, Rb-Ser(P)-795, Cdk2, Cdk4, CyclinD1, GFP, Thr(P), perifosine, and human recombinant IGF-1 were from Cell Signaling Technology. Anti-MCM7, Flag, and V5 antibodies were purchased from Santa Cruz. Antibody for RACK1 was from BD Transduction Laboratories. Antibodies for MCM4, MCM6, Cdt1, p27, E2F1, and Histone 3 were from Proteintech Group. Anti-Ser(P) antibody was from Millipore.

### Plasmids, siRNAs, and transfection

Control non-targeting siRNA and siRNAs targeting human RACK1 and Akt were from Santa Cruz. pEGFP-N1-RACK1 was a gift from Anna Huttenlocher (Addgene plasmid # 41088) [57]. pLenti6/V5-DEST-MCM7 was a gift from Lynda Chin (Addgene plasmid # 31212) [58]. 1477 pcDNA3-Flag-HA-Akt1 was a gift from William Sellers (Addgene plasmid # 9021) [59]. pEGFP-N1 was subcloned from pEGFP-N1-RACK1 and pcDNA-Flag-HA was subcloned from pcDNA-Flag-HA-Akt1. pGBKT7-MCM7 and pGADT7-RACK1 were kindly provided by Dr. Jianhua Luo, University of Pittsburgh. Cells were transfected using Lipofectamine 3000 (Invitrogen).

### Immunoprecipitation and immunoblotting

Cells were harvested in cell lysis buffer (50 mM Tris, pH 7.5, 150 mM NaCl, 1% Triton X-100, 1 mM EDTA, 2.5 mM sodium pyrophosphate, 1% Na_3_VO_4_, 1 mM PMSF, 0.5 μg/mL leupeptin, 10 μg/mL aprotinin, 1 mM phosphatase inhibitors). Cell lysates were incubated on ice for 30 min and then centrifuged at 12000 rpm for 20 min. The resulting supernatants were collected and assayed in immunoblotting experiments. For immunoprecipitation experiments, the relevant antibodies were added (1 μg/1 mg lysates) and incubated at 4°C overnight. Fifty μL of protein A/G beads were then added and incubated for an additional 4 h at 4°C. The beads were collected, washed 5 times with lysis buffer, and resuspended in 1 × SDS sample buffer. The samples were then separated by SDS-PAGE and immunoblotted using the relevant antibodies.

### Chromatin association assay

The chromatin association assay was performed as previously described [37]. Cells were lysed in 1 mL of Buffer A (15 mM NaC_2_H_3_O_2_, 110 mM KC_2_H_3_O_2_, 2 mM MgC_2_H_3_O_2_, 0.5 mM EGTA, and 20 mM HEPES, pH 7.3). DTT and digitonin were then added to the cell suspension at final concentrations of 2 mmol/L and 50 mg/mL, respectively, and suspensions were placed on ice for 10 min. The suspension was then centrifuged at 1500 g for 10 min at 4°C. Pelleted nuclei were resuspended in hypotonic buffer (Buffer B: 1 mM HEPES, pH 7.5, 0.5 mM EDTA supplemented with 0.5% NP40). The nuclear suspensions were then placed on ice for 15 minutes and laid on top of a 10-mL sucrose cushion (100 mM sucrose and 0.5 mM Tris-HCl, pH 8.5) and centrifuged at 3500 g for 10 minutes at 4°C. The chromatin pellets were suspended in 0.25 mM EDTA (pH 8.0) and sonicated for 10 seconds two times per sample to produce a chromatin-rich supernatant fraction. Proteins in these fractions were quantitated and analyzed using SDS-PAGE.

### Colony formation assay

The A549 and H460 cells were plated in 40-mm dishes 24 h after transfection (1000 per dish) and incubated for 12 days. Medium containing 10% fetal bovine serum was replaced every 4 days. On day 12, the plates were washed with PBS, stained with hematoxylin, and the number of colonies with more than 50 cells was counted.

### 3-(4,5-Dimethylthiazol-2-yl)-2,5-diphenyltetrazolium bromide (MTT) assay

Cells were plated in 96-well plates at a concentration of 3000–5000 cells per well 24 h after transfection. To measure cell growth, 20 μL of 5 mg/mL MTT was added to the media and samples were incubated for 4 h at 37°C. The media were then removed, 200 μL of DMSO was added to dissolve the generated deposits, and the absorbance at 490 nm was measured using an automatic microplate reader. The measurement process was performed every 24 h for 5 days to generate a cell growth curve.

### Immunofluorescence

Twenty-four hours after they were plated on covered slides, cells were washed 3 times with ice-cold PBS and fixed in 4% paraformaldehyde for 20 min at room temperature. After two PBS washes, the cells were blocked with 3% BSA with 0.2% Triton X-100. Cells were then stained with primary antibodies diluted in 3% BSA overnight at 4°C. After 3 washes in PBS, cells were incubated for 2 h with the secondary donkey anti-mouse (fluorescein isothiocyanate conjugated, 1:300) or goat anti-rabbit (tetramethylrhodamine-isothiocyanatee conjugated, 1:300) antibodies, as appropriate. Nuclei were counterstained with propidium iodide (PI, 50 μg/mL, Sigma).

### Flow cytometry

For DNA content analysis, cells were fixed in 70% ethanol overnight at 4°C and then washed with PBS containing 1% BSA. Cells were incubated in 50 μg/mL propidium iodide and 100 μg/mL RNase A for 30 min, and 10000 cells per sample were analyzed using a FACSCalibur cytometer (BD Biosciences).

### Yeast two-hybrid screening

The fusion protein pGBKT7-MCM7 contained 719 amino acids from MCM7 and 219 amino acids from bait domain [37]. The construct was transformed into One ShotTM competent cells (Invitrogen, Carlsbad, CA). pAD-RACK1 was constructed in pACT2 in 0.5 mL of polyethylene glycol/LiAc and incubated at 30°C for 30 minutes. After this initial incubation with plasmid DNA, the cell solution was combined with 20 mL of DMSO and incubated for 15 minutes at 42°C. The cells were pelleted, resuspended in 1 mL YPD medium, and shaken at 30°C for 40 minutes. The transformed cells were then pelleted, resuspended in 0.5 mL 0.9% NaCl, and plated onto the appropriate SD agar plate. The transformants were first plated on low stringency SD-Leu/-Trp and medium stringency SD-Leu/-Trp/-His plates. The colonies that grew on those plates were then subjected to the β-galactosidase assay as previously described for 24 and then allowed to grow further on the high stringency SD-Ade/-His/-Leu/-Trp plate.

### Patients and specimens

This research was approved by the Human Research Ethics Committee of China Medical University, which is accredited by the National Council on Ethics in Human Research. All patients signed informed consents and were closely monitored during follow-up observations. None of the patients received chemotherapy or radiotherapy prior to operations.

### Primary tumor specimens

Primary NSCLC and matched normal lung tissue specimens were obtained from 28 patients who underwent surgical resection at the First Affiliated Hospital of China Medical University between 2010 and 2011. Samples were cut from the resected lungs immediately after removal, frozen in liquid nitrogen, and then stored at −80°C.

### Paraffin sections

The following tissue chips were purchased from Shanghai Core Super Biological Technology Co., LTD: lung cancer 120 point OD-CT-DgLug01-007 (20 adenocarcinoma cases, 10 squamous cell carcinoma cases, 10 adenosquamous carcinoma cases, 10 large cell lung cancer cases, 10 small cell lung cancer cases), lung adenocarcinoma 150 point OD-CT-RsLug01-008 (75 adenocarcinoma cases), and lung squamous cell carcinoma 150 point OD-CT-RsLug01-009 (75 squamous cell carcinoma cases). One hundred fifty NSCLC cases contained detailed information and prognostic information (78 adenocarcinoma cases, 72 squamous cell carcinoma cases).

### Immunohistochemical analysis

Briefly, tissue samples were deparaffinized in xylene and endogenous peroxidase activity was blocked with 4% H_2_O_2_. The sections were incubated with RACK1-specific mouse polyclonal antibody (1:100 dilution) or MCM7-specific rabbit polyclonal antibody (1:50 dilution) overnight at 4°C, incubated with biotin-labeled secondary antibodies (Ultrasensitive; Fuzhou MaiXin Biotechnology Development Co., Ltd., Fujian, China) at 37°C for 30 min, and then incubated with diaminobenzidine for coloration.

### RNA extraction and RT-PCR

Total RNA was isolated from NSCLC patient tissues using TRIzol reagent (Invitrogen, USA) according to the manufacturer's instructions. The cDNA templates were as follows: RACK1 (357 bp) fragment with primers 5′-TGAGTGTGGCCTTCTCCTCT-3′ (forward) and 5′-AAAGGTGTTTGCCTTCGTTG-3′ (reverse); MCM7 (382 bp) fragment with primers 5′-ACCGAGACAATGACCTACGG-3′ (forward) and 5′-CTAGCTGTCTGCCCCTTGTC-3′ (reverse); and internal control gene β-actin (345 bp) with primers 5′-CTCCATCCTGGCCTCGCTGT-3′ (forward) and 5′-GCTGTCACCTTCACCGTTCC-3′ (reverse). Thermal cycling reactions were performed using standard conditions with annealing temperatures of 58.0°C (for RACK1), 60.0°C (for MCM7) and 55.5°C (for β-actin), respectively, for 40 cycles. The products were electrophoresed on 1.2% agarose gels and then semi-quantified using the Gel-pro Analyzer image analysis software package (Media Cybernetics, USA).

### Statistics

Immunohistochemistry results were analyzed using the chi-square test and Spearman rank correlation. Kaplan-Meier survival analyses were carried out and compared using the log-rank test. Differences between groups were compared using two-tailed Student's *t-test*; *p* values < 0.05 (*) or < 0.01 (**) were considered statistically significant.

## Abbreviations

MCM7: Miniature chromosome maintenance 7
RACK1: Receptor for activated C kinase 1
ORC: Origin recognition complex
NSCLC: Non-small-cell Lung Cancer
